# Supplementary material for: SCENIC+: single-cell multiomic inference of enhancers and gene regulatory networks
Source: Nat Methods. 2023 Jul 13;20(9):1355–67. doi: 10.1038/s41592-023-01938-4 (PMC10482700; doi:10.1038/s41592-023-01938-4)
Supplement: Supplementary file 1 — Supplementary Notes 1–6. [file 41592_2023_1938_MOESM1_ESM.pdf]

nature methods

Article https://doi.org/10.1038/s41592-023-01938-4

SCENIC+: single-cell multiomic inference of enhancers and gene regulatory networks

In the format provided by the authors and unedited

Table of Contents

Supplementary Notes *2*

Supplementary Note 1: The SCENIC+ workflow **2**

Supplementary Note 2: The SCENIC+ motif collection **14**

Supplementary Note 3: pycisTarget: Motif enrichment databases and analysis algorithms **15**

Supplementary Note 4: Benchmark of GRN inference methods **17**

Supplementary Note 5: Spatial projection of SCENIC+ regulons in the mammalian cortex **19**

Supplementary Note 6: Predicting repressive interactions using SCENIC+ **20**

Supplementary References *23*

Supplementary Notes

Supplementary Note 1: The SCENIC+ workflow

The SCENIC+ workflow consists of three main steps, performed with three linked Python modules: 1) unsupervised identification of enhancers with shared accessibility patterns from scATAC-seq data (*pycisTopic*), 2) prediction of TF binding sites via motif enrichment analysis (*pycisTarget*) and 3) prediction of eGRNs combining TF expression, TF binding sites, region accessibility and gene expression (*SCENIC+*). The minimal input for SCENIC+ is a gene expression matrix with cell type annotation and a corresponding scATAC-seq fragments file. The two latter can be replaced by a precompiled matrix with fragment counts and precomputed peak coordinates.
The three Python modules include detailed tutorials to facilitate their use for standalone analyses. Links to the tools, SCENIC+ code and tutorials are available at: scenicplus.readthedocs.io.

PycisTopic

Pycistopic is an improved Python-based version of our Bayesian framework cisTopic[1], which exploits a topic modelling technique called Latent Dirichlet Allocation (LDA)[2]. This unsupervised approach simultaneously clusters cells and co-accessible regions into regulatory topics. Outside the SCENIC+ framework, pycisTopic can be used to analyze independent scATAC-seq data as well. PycisTopic is available at https://github.com/aertslab/pycisTopic , with full documentation and tutorials available at pycistopic.readthedocs.io The full pycisTopic pipeline consists of the following steps (*indicates those required for the SCENIC+ workflow, ** indicates those recommended for the SCENIC+ workflow):

- **Consensus peak calling*:** PycisTopic will first create a set of consensus peaks across all cells by calling and merging peaks on pseudobulk ATAC-seq profiles per cell type. First, pseudobulk fragment bed files per cell type are generated utilizing the fragments file and cell type annotations provided by the user. In a second step, peaks are called using MACS2[3], with parameters –format BEDPE (as we are providing fragments bed files as input) and –keep-dup all --shift 73 --ext_size 146, as recommended for scATAC-seq data. To derive a set of consensus peaks, an iterative overlap peak merging procedure is used as described in Corces et al.

2018[4]. First, each summit is extended a `*peak_half_width*` (by default, 250bp) in each direction and less significant peaks are iteratively filtered out. During this procedure peaks will be merged and depending on the number of peaks included into them, different processes will happen: 1) 1 peak: The original peak will be kept, 2) 2 peaks: The original peak region with the highest score will be kept and 3) 3 or more peaks: The original region with the most significant score will be taken, and all the original peak regions in this merged peak region that overlap with the significant peak region will be removed. The process is repeated with the next most significant peak (if it was not removed already) until all peaks are processed. This procedure will happen twice, first for each pseudobulk peak, and after peak score normalization to process all peaks together. We recommend using this set of regions downstream, as we have observed that using pseudobulk peaks improves signal compared to using bulk peaks across the whole population (specially for rare cell types, whose signal may be confused by noise while using the merged ATAC-seq profile of the whole population). In case of independent scATAC-seq data, the cell annotation can also be obtained from alternative methods, such as a preliminary clustering analysis using a predefined set of genome-wide regions/peaks (e.g. SCREEN[5]) as input to identify cell populations.

- **QC analysis and cell selection**:** PycisTopic computes QC metrics at the sample-level and the barcode-level. Sample-level statistics can be used to assess the overall quality of the sample, while barcode level statistics can be used to differentiate good quality cells versus the rest.

Sample-level statistics include:

- **Barcode rank plot**: The barcode rank plot shows the distribution of non-duplicate reads and which barcodes were inferred to be associated with cells. A steep drop-off ('knee') is indicative of good separation between the cell-associated barcodes and the barcodes associated with empty partitions.
- **Insertion size**: ATAC-seq requires a proper pair of Tn5 transposase cutting events at the ends of DNA. In the nucleosome-free open chromatin regions, many molecules of Tn5 will fragment the DNA; around nucleosome-occupied regions, Tn5 can only access the linker regions. Therefore, in a good ATAC-seq library, one should expect to see a sharp peak at the <100 bp region (open chromatin), and a peak at ~200bp region (mono-nucleosome), and other larger

peaks (multi-nucleosomes). A clear nucleosome pattern indicates a good quality of the experiment.

- **Sample TSS enrichment**: The TSS enrichment calculation is a signal to noise calculation. The reads around a reference set of TSSs are collected to form an aggregate distribution of reads centered on the TSSs and extending to 1,000 bp in either direction (for a total of 2,000bp). This distribution is then normalized by calculating a fold change compared to 100bp at either side of the flanks of the distribution.
- **FRiP distribution**: Fraction of all mapped reads that fall into the called peak regions, i.e. usable reads in significantly enriched peaks divided by all usable reads. A low FRIP indicates that many reads form part of the background, and so that the data is noisy.
- **Duplication rate**: A fragment is considered “usable” if it uniquely maps to the genome and remains after removing PCR duplicates (defined as two fragments that map to the same genomic position and have the same unique molecular identifier). The duplication rate serves to estimate the number of usable reads per barcode. High duplication rates may indicate over-sequencing or lack of fragments after transposition and encapsulation.

Barcode-level statistics include:

- **Total number of (unique) fragments per cell-barcode**
- **TSS enrichment per cell-barcode**: The normalized coverage at the TSS position for each barcode (average +-10bp from the TSS). Noisy cells will have a low TSS enrichment.
- **FRiP per cell-barcode**: The fraction of reads in peaks for each barcode. Noisy cells have low FRIP values. However, this filter should be used with nuance, as it depends on the quality of the original peaks. For example, if there is a rare population in the sample, its specific peaks may be missed by peak calling algorithms, causing a decrease in their FRIP values.

- **Count matrix generation*:** PycisTopic can generate a fragment count matrix from the fragments files, a set of regions (preferably, consensus regions as previously explained) and a list of high quality cells. Alternatively, a precomputed count matrix can also be used as input. In this step a cisTopic object will be created, including the fragment counts, path/s to the fragments files (if used to generate the count matrix) and cell/region metadata.

- **Doublet identification:** The fragment count matrix can be used as input for Scrublet[6] (v0.2.3), By default, and when dealing with 10x data sets, the expected doublet rate is set to 10%.
- **Topic modelling algorithms and model selection*:** PycisTopic implements two algorithms for topic modelling, serial LDA with a Collapsed Gibbs Sampler (as implemented in the lda module) and Mallet, which allows to parallelize the LDA model estimation. The same default parameters as in cisTopic[1] are used. Following metrics for model selection are included in pycisTopic:
  - **Mimno_2011**: Uses the average model coherence as calculated by Mimno et al (2011)[7]. To reduce the impact of the number of topics, the average coherence based on the top values is used. Better models have a higher coherence.
  - **Log-likelihood**: Uses the log-likelihood in the last iteration as calculated by Griffiths and Steyvers (2004)[8], as used in cisTopic. Better models have a higher log-likelihood.
  - **Arun_2010**: Uses a density-based metric as in Arun et al (2010)[9] using the topic-region distribution, the cell-topic distribution and the cell coverage. Better models have a lower score for this metric.
  - **Cao_Juan_2009**: Uses a divergence-based metric as in Cao Juan et al (2009)[10] using the topic-region distribution. Better models have a lower score for this metric.

The best model (i.e. the one with the optimal number of topics) is the model with the smallest number of topics where coherence (Minmo_2011) and Log-likelihood are maximized and Arun_2010 and Cao_Juan_2009 are minimized.

- **Dimensionality reduction and batch effect correction**:** Cells (or regions) can be clustered using the leiden algorithm and embedded using dimensionality reduction with UMAP and TSNE using the cell-topic (or topic region distributions). In addition, harmonypy (v0.0.5) can be used on scaled cell-topic distributions to correct for batch effect between samples (see mouse cortex analysis). When working with single cell multiome data, it is possible to co-cluster and reduce dimensionality using both the scRNA-seq and scATAC-seq data by using UMAP to build fuzzy simplicial sets (similar to KNN graphs).
- **Topic binarization and QC*:** To perform motif analysis (and other downstream steps) topics have to be binarized into region sets rather than continuous distributions. Several binarization methods are included in pycisTarget (applicable

for topic-region and cell-topic distributions): *'otsu'* (Otsu, 1979)[11], *'yen'* (Yen et al., 1995)[12], *'li'* (Li & Lee, 1993)[13], *'aucell'* (Van de Sande et al., 2020)[14] or 'ntop' (Taking the top n regions per topic). Otsu and Yen's methods work well for topic-region distributions; however, for some downstream analyses, it may be convenient to use 'ntop' to have balanced region sets (e.g. training of classification models). By default, pycisTopic uses Otsu thresholding for binarization, as it results in the largest number of regions per topic while limiting the amount of "noise" for motif enrichment analysis. For cell-topic distributions, we recommend using the AUCell method. In addition, pycisTopic includes new metrics to assess topic quality:

- **Number of assignments and regions/cells per binarized topic.**
- **Topic coherence (Mimno et al., 2011)**[7]: Measures to which extent high scoring regions in the topic are co-accessible in the original data. If it is low, it indicates that the topic is rather random. Better topics have a higher score.
- **The marginal topic distribution**: Indicates how much each topic contributes to the model. Better topics have a higher score.
- **The gini index**: Value between 0 and 1, that indicates the specificity of topics (0: General, 1: Specific)

- **Drop-out imputation*:** Thanks to the probabilistic nature of topic modelling, drop-outs can be imputed by multiplying the cell-topic and topic-region distributions, resulting in a matrix with the probabilities of each region in each cell.
- **Calculation of Differentially Accessible Regions (DARs)*:** Using the imputed fragment matrix Differentially Accessible Regions, or DARs can be calculated. Briefly, a Wilcoxon rank sum test is performed for user specified contrasts. By default, a DAR is defined as a region with LogFC > 0.5 and benjamini hochberg adjusted p values < 0.05.
- **Gene activity and Differentially Accessible Genes (DAGs):** The gene activity recapitulates the overall accessibility values in a space around the gene. Differentially Accessible Genes (DAGs) can be derived from this matrix. The user can select among different options:
  - **Search space**: The user can choose whether the search space should include other genes or not (*use_gene_boundaries*), and the minimum and maximum distance it should have (*upstream* and *downstream*). Promoters can be excluded from the calculations, as they are usually ubiquitously accessible.

- **Distance weight**: The parameters weights the impact of distance when inferring region to gene weights as an exponential function. The user can control whether this weight should be used (*distance_weight*) and the effect of the distance (*decay_rate*). In addition, the user can choose from which distance to the gene body this weight is applied (*extend_gene_body_upstream* and *extend_gene_body_downsstream*)
- **Gene size weight**: Large genes may have more peaks by chance. The user can optionally apply a weight based on the size of each gene (*gene_size_weight*), which by default is dividing the size of each gene by the median gene size in the genome. Alternatively, the user can also use *average_scores* which will calculate the gene activity as the mean weighted region accessibility of all regions linked to the gene.
- **Gini weight**: This weight will give more importance to regions that are highly specific (*gini_weight*).

- **Label transfer:** PycisTopic includes wrappers for several label transfer methods using annotated scRNA-seq and the gene activity matrix. The methods available for label transferring are: '*ingest*'[15], '*harmony*'[16], *'bbknn'*[17], *'scanorama'*[18] and *'cca'*. Except for ingest, these methods return a common coembedding and labels are inferred using the distances between query and reference cells as weights.
- **pyGREAT:** pycisTopic makes GREAT (Genomic Regions Enrichment of Annotations Tool)[19] analysis automatic by constructing a HTTP POST request according to an input region set and automatically retrieving results from the GREAT web server.
- **Signature enrichment:** Given epigenomic signatures are intersected with the regulatory regions in the dataset and summarized into region sets. Using the imputed fragment matrix, all regions in each cell are ranked and the cell-specific rankings are used as input for AUCell. By default, we use 5% of the total number of regions in the dataset as a threshold to calculate the AUC.
- **Export to loom files**:** PycisTopic allows to export cisTopic object to loom files compatible with Scope for visualization[20] and SCopeLoomR, for importing pycisTopic analyses into R.

PycisTarget

PycisTopic unsupervisedly identifies groups of co-accessible regions (cis-regulatory topics) as well as cell type specific enhancers (DARs). These regulatory programs are used in the second step of SCENIC+, in which TFs and their potential target regions (i.e. cistromes) are identified using motif enrichment analysis. For this purpose, we have developed pycisTarget, a motif enrichment suite that combines different motif enrichment approaches such as cisTarget[21–23] and Homer[24]; and a novel approach to compute Differentially Enriched Motifs between sets of regions called DEM. Pycistarget is available at https://github.com/aertslab/pycistarget , with full documentation and tutorials available at pycistarget.readthedocs.io.

- **Homer:** PycisTarget includes a wrapper to run Homer's *findMotifsGenome.pl*, allowing the identification of known and *de novo* motifs (by default using default Homer parameters). For identifying cistromes for each motif, found motifs are used as input for *homer2 find*. Known motifs are annotated according to the motif annotation in the SCENIC+ motif collection. To annotate *de novo* motifs, Tomtom[25] is run with the SCENIC+ motif collection to identify the closest match, allowing to transfer its annotation to the *de novo* motif when specified. To form TF cistromes, motif-based cistromes are combined based on the TF annotations.
- **Generation of cisTarget databases:** cisTarget and DEM require ranking and score-based databases, respectively, with regions as rows, motifs (or motif clusters) as columns, and scores or ranking values of these scores, as values. For this, we have developed an adapted version of Cluster-Buster[26], which is now 2 times faster. Cluster-Buster uses Hidden Markov Models (HMMs) to score clusters of motifs (i.e., Cis-Regulatory Modules (CRM)) given a set of regions. This implementation is available at https://resources.aertslab.org/cistarget/programs/cbust. The source code for our cluster-buster implementation is available at https://github.com/ghuls/cluster-buster/tree/change_f4_output. Briefly, given a motif collection (in cb format) and a set of regions, Cluster-Buster is run using each motif across all regions. When dealing with motif clusters, Cluster-Buster uses all motif variations by implanting each motif as a hidden state in a HMM, and each candidate sequence receives a log-likelihood ratio (LLR) score per motif cluster (i.e. CRM score). A scores database is first generated by taking the highest CRM score per sequence. A ranking database is then generated by ranking, for each motif, all the regions by decreasing motif score. The code and documentation to generate these databases

is available at https://github.com/aertslab/create_cisTarget_databases. The motif collection to generate custom databases is available at https://resources.aertslab.org/cistarget/motif_collections/. We provide precomputed motif databases using predefined sets of regulatory regions for hg38, mm10 (using SCREEN regions[5]) and dm6 (using cisTarget regions, defined by partitioning the entire non-coding *Drosophila* genome based on cross-species conservation) at: https://resources.aertslab.org/cistarget/databases/. cisTarget databases can also be generated using genomic tracks, for instance from TF ChIP-seq. To generate track databases, a bed file indicating each genomic region and the average signal (e.g. as output from *bigWigAverageOverBed*) has to be provided. Regions will then be sorted per track in decreasing order based on these scores. In the case of TF ChIP-seq tracks, tracks are linked to the TF that was targeted in the ChIP-seq experiment.

- **cisTarget:** pycisTarget implements the ranking based motif enrichment method cisTarget[21–23]. Briefly, genomic regions (i.e. consensus peaks, or predefined regions from SCREEN) are first scored using a motif collection with Cluster-Buster, as previously described. These regions will be ranked in decreasing order based on their score for each motif. The input regions are intersected with regions in the database (with at least 40% overlap). cisTarget uses a recovery curve approach (for each motif), in which a step is taken in the y-axis when as region in the motif ranking (x-axis) is found in the region set. The Area Under the Curve for each motif is normalized based on the average AUC for all motifs and their standard deviation, resulting in a Normalized Enrichment Score (NES) that is used to quantify the enrichment of a motif in a set of regions:

$$NES = \frac{AUC - mean(AUC)}{sd(AUC)}$$

With:

$mean(AUC)$	The average AUC values across all motifs
$sd(AUC)$	The standard deviation of AUC values across all motifs

By default, motif that obtain a NES above 3.0 are kept. To obtain the target regions for each motif (motif-based cistrome) the regions at the top of the ranking (leading edge) are retained. The top of the ranking is defined by an automated thresholding method

that retains regions with a ranking below the rank at max, which is defined by the following formula:

$$RankAtMax = \max\left(rcc_{motif} - \left[\mu\left(rcc_{all\ motifs}\right) + 2 \cdot SD\left(rcc_{all\ motifs}\right)\right]\right)$$

With:

rcc_{motif} the recovery curve of the motif of interest.

$\mu\left(rcc_{all\ motifs}\right)$ the average recovery curve over all motifs.

$SD\left(rcc_{all\ motifs}\right)$ the standard deviation of the recovery curve over all motifs.

To obtain target region for each TF, the motif-based cistromes of motifs annotated to the TF are merged.

- **DEM:** DEM performs a Wilcoxon test between scores in foreground and background region sets to assess motif enrichment. Briefly, genomic regions (i.e. consensus peaks, or predefined regions from SCREEN[5]) are first scored using a motif collection with Cluster-Buster, as previously described. Regions in the input region sets are intersected with regions in the database (with at least 40% overlap), and a Wilcoxon rank sum test is performed between CRM score distributions in the two groups. By default, motifs adjusted p-value < 0.05 (Bonferroni) and LogFC > 0.5 are kept. A cistrome for each motif is generated by simultaneously optimizing precision and recall to separate foreground from background regions or using a predefined CRM threshold. To obtain the target regions for each TF, the motif-based cistromes of motifs (annotated to that TF are combined.

Within the SCENIC+ workflow, motif enrichment is performed by default in binarized topics and DARs calculated by pycisTopic, using cisTarget and DEM (including and excluding promoters from the region sets). By default, DEM is run using topics or DARs as foreground and 500 regions in other topics/DARs as background (with the same proportion of promoters as in the foreground). Additional region sets (e.g. DARs derived from specific contrasts instead of using all populations as background) can be easily added. Cistromes derived from all the motif enrichment analysis are merged by TF to generate a final set of TF-region cistromes.

SCENIC+

eGRNs are predicted in the final step of SCENIC+. This step requires as input: the gene expression matrix, the imputed accessibility matrix (from pycisTopic) and the TF

cistromes (from pycisTarget). Input data can be single-cell multiome or paired scRNA-seq and scATAC-seq in matching populations (see below). This final step consists of the following steps:

- **Generating pseudo-multiome data (in case of non-multiome data):** To generate pseudo multiome data, cells must be annotated by common labels in both data modalities (single-cell chromatin accessibility and gene expression). Pseudo multiome cells are generated by sampling a predefined number of cells from each data modality, within the same cell type annotation label, and averaging the raw gene expression and imputed chromatin accessibility data across these cells to create a multiome meta cell containing data of both modalities. By default, the number of meta cells generated for each cell type annotation label is set as such that each cell is included in a meta cell on average twice.
- **Calculating TF-to-gene and region-to-gene relationships:** TF-to-gene relationships are calculated as described in[14,27]. Briefly, the Arboreto python package (v0.1.6) is used to calculate TF-to-gene importance scores for each TF and each gene, given a list of known TFs and the raw gene expression matrix. By default, Gradient Boosting Machine regression is used. Pearson correlation is used to separate positively correlating from negatively correlating relationships (resp. correlation coefficient above 0.03 or below –0.03 by default). The TF itself is not included in the initial TF-gene relationship calculation (otherwise it would skew the importance scores of the other genes). Therefore, in order to be able to infer autoregulation (TFs regulating their own expression) the importance score of the TF itself is set to the maximum importance score across all genes added with an arbitrary small value of 1E-5, in order to put the TF at the top of its own ranking. Region-to-gene relationships are calculated for each gene by considering all regions within a search space surrounding that gene. By default, a search space of a minimum of 1kb and a maximum of 150kb upstream/downstream of the start/end of the gene or the promoter of the nearest upstream/downstream gene is used. By default, the promoter of the gene is considered as the TSS of the gene +/- 10 bps. For each consensus peak in the search space of each gene region-to-gene importance scores are calculated using the Arboreto python package (v0.1.6) using the imputed accessibility of the regions as predictors for the raw gene

expression counts and Gradient Boosting Machine regression (by default). Spearman rank correlation (SciPy v1.8.1) is used to separate positively correlating from negatively correlating relationships (resp. correlation coefficient above 0.03 or below –0.03 by default). Before eGRN building, region-to-gene relationships are binarized using multiple methods. By default, the 85th, the 90th and the 95th quantile of the region-to-gene importance scores, the top 5, 10, and 15 regions per gene based on the region-to-gene importance scores and a custom implementation of the BASC[28] method on the region-to-gene importance scores is used.

- **eRegulon creation:** We define an eRegulon as a TF together with its target regions and genes. To generate this, information from gene expression, region accessibility and motif enrichment are combined. For each TF, TF-region-gene triplets are generated by taking all regions that are enriched for a motif annotated to the TF and all genes linked to these regions, based on the binarized region-to-gene links (see "Calculating TF-gene and region-gene relationships"). However, we only want to include genes, and the regions linked to them, in the final eRegulon if they are significantly co-expressed with the TF. To determine this, Gene Set Enrichment Analysis (GSEA) is used. Here, all genes are ranked based on the TF-gene importance scores and an enrichment analysis of the set of genes in the triplet compared to the overall ranking of the genes is computed, using the *gsea_compute* function from the GSEApy python package (v0.10.8). Finally, only the genes at the top of the ranking, known as the leading edge are retained in the final eRegulon. This analysis is run separately for TF-gene and region-to-gene relationships with positive and negative correlation coefficients, for a total of four GSEA runs. By default, eRegulons with less than 10 predicted target genes or obtained from region-to-gene relationships with a negative correlation coefficient are discarded.
- **eRegulon enrichment:** Target regions and genes of each eRegulon are used separately together with regions and genes ranked based on imputed accessibility and raw gene expression counts in each cell as input for AUCell[27], using the ctxcore python package (v. 0.1.2.dev2+g1ffcf0f). By default, 5% of the total number of regions or genes in the dataset are used as threshold to calculate AUC values. High quality regulons are then selected based on the

correlation between region based and gene-based AUC values (by default 0.4) and/or AUC values and TF expression.

- **eRegulon dimensionality reduction:** The eRegulon enrichment scores for regions and genes are normalized for each cell and used as input into the UMAP, tSNE or PCA from the python package umap (v0.5.2), fitsne (v1.2.1) or Scikit-learn (v0.24.2) respectively.
- **eRegulon specificity scores:** eRegulon specificity scores are calculated using the RSS algorithm as described in[14,29] using target region or target gene eRegulon enrichment scores as input. The Regulon Specificity Score (RSS) is used to identify marker regulons that differentiate between clusters or cell types. We use the RSS for plotting regulon enrichment (as it normalizes the AUCell values) and to prioritize regulons per cell type (to select the most specific regulons for plotting, or to prioritize the differentiation velocities).
- **Triplet ranking:** all TF-region-gene triplets, as identified by SCENIC+, are ranked by generating the aggregated ranking of the TF-to-gene score, region-to-gene score and TF-to-region score. The TF-to-gene and region-to-gene scores are defined as the Gradient Boosting Machine regression importance scores for predicting gene expression from resp. TF expression and region accessibility across all cells. The TF-to-region score is defined as the best ranked position of the region across the ranks of all motifs annotated to the TF of interest. Ranks are aggregated as described in[30].
- **Export to loom files:** To visualize SCENIC+ results in SCope[20] a chromatin accessibility- and gene expression-based loom file containing the eRegulons with target regions/genes and eRegulon enrichment scores for target regions/genes is generated, using the LoomXpy python package (v0.4.1; https://github.com/aertslab/LoomXpy). In addition, loom files can also be used to import data into R via the SCopeLoomR package.
- **Visualization in the UCSC genome browser:** To visualize SCENIC+ results in the UCSC genome browser a UCSC interaction file, containing region-to-gene links color coded by region-to-gene importance scores or correlation coefficients, and a bed file, containing genomic coordinates of eRegulon target regions is generated. The UCSC interact file and the bed file are converted to the bigInteract and bigBed format using the bedtobigbed program (v2.7) from

UCSC. These can be uploaded to the UCSC genome browser along with pseudobulk BigWig files.

- **Network visualization:** Enhancer GRNs can be visualized using networkx (v2.7.1) concentrical and Kamada-Kawai layouts, with customized features for nodes (size, shape, color, transparency) and edges (stroke, color, transparency). Figures can be generated with networkx (v2.7.1) or interactively with pyvis (v0.1.3.1). In addition, SCENIC+ also can export networks to Cytoscape (v3.9.0). The SCENIC+ style for Cytoscape is available at: https://github.com/aertslab/scenicplus/tree/main/cytoscape_styles.

Supplementary Note 2: The SCENIC+ motif collection

The SCENIC+ motif collection includes more than 49,504 motifs from 29 motif collections (Supplementary Table 3), with curated TF motif annotations based on direct evidence and orthology across species for human, mouse and fly. In order to account for motif redundancy (i.e. the same or a very similar version of the same motif can be found in more than one of these collections), we implemented an approach to create non-redundant (or clustered) motif collections using a two-step clustering strategy. First, identical PWMs across collection (after rescaling) were merged, resulting in 34,524 motifs. A matrix with motif-to-motif similarity values was computed using Tomtom (MEME v5.4.1), and motifs with equal length and q-value < 10^{-40} were merged, resulting in 32,766 motifs (unclustered motif collection). For clustering, motifs that are similar to at least another motif (q-value < 10^{-5} (n=11,526)) were used, while the remaining were kept as unique motifs, or singlets (n=9,685). Dimer motifs (1,265) were excluded from the clustering, as well as motifs from factorbook and desso, as they do not have direct annotations since they are derived from AI models. Motifs with an Information Content below 5 were also excluded. We converted the motif similarity value matrix to -log10(Tomtom similiarity)+10^{-45}. Seurat (v4.0.3) was used to normalize, scale and perform PCA on this matrix. Using 100 PCs, Leiden clustering with resolution 25 was performed, resulting in 199 clusters. STAMP was run (v1.3; using the -cc -sd –chp option) to refine clusters resulting in 1,985 clusters. For each cluster, STAMP's consensus was used as the logo. The TF annotation of a cluster was inferred by merging the TF annotations (direct and orthology) of all its members.

Overall, the clustered motif collection contains 9,685 singlets, 1,265 dimers and 1,985 clusters (with a mean of 5.8 motifs per cluster).

The SCENIC+ motif collection contains 8,384, 8,045, 958 annotated clusters for 1,553, 1,357 and 467 TFs (with an average of 5, 6, 2 motifs per TF) for human, mouse, and fly; respectively. Importantly, motifs are not only annotated based on direct TF-motif experimental evidence; but also based on orthology (by downloading gene orthologs for the selected species from Ensembl Biomart 105), which permits the incorporation of annotations based on experiments in different species. In fact, 433 mouse TFs are only found via orthology, augmenting TF-annotations by 47%, as more experiments have been performed in human systems than in mouse.

We provide all PWMs and clusters of the SCENIC+ motif collection for the creation of custom databases (with the exemption of the licensed Transfac Pro PWMs) at https://resources.aertslab.org/cistarget/motif_collections/, and precomputed databases using genomic regions (SCREEN for mouse and human, and cisTarget regions for fly) and updated gene-based databases for SCENIC at https://resources.aertslab.org/cistarget/databases/.

Supplementary Note 3: pycisTarget: Motif enrichment databases and analysis algorithms

To perform motif enrichment analysis two databases are generated, a score-dbased database and a ranking-based database, with regions as rows and (cluster of) motif(s) as columns (Fig. 1d). Regions in the databases can be either dataset specific consensus peaks or a pre-defined set of regions (for example, we provide precomputed databases on the ENDODE's SCREEN regions[5]). In the latter case, the predefined regions are intersected with dataset specific regions internally, however this approach usually results in the loss of certain regions that are not included in the SCREEN database. To generate the databases, regions are first scored for each (cluster of) motif(s) using Cluster-Buster[26]. These scores are log-likelihood ratios (LLRs) capturing the probability of the sequence given the PWM model, over the probability of the sequence given the background model. Cluster-Buster uses a Hidden Markov Models (HMM), which permits to use every motif in a PWM cluster as one of the hidden states in the HMM. This way a score is generated for each region

and each PWM cluster, while using all the PWM variation present in each cluster (Fig 1d). The ranking matrix is obtained by ranking for each motif the regions based on their scores (in decreasing order).

Next, motif enrichment across a set of regions is calculated using two methods, namely DEM and cisTarget (Fig. 1d, see Methods). The DEM algorithm, which uses the score-based database, compares LLRs of the set of regions (i.e. foreground) to a chosen background set using a Wilcoxon rank sum test. The cisTarget algorithm, which utilizes the ranking-based database, uses a recovery curve approach (for each motif), in which a step is taken on the y-axis when the region in the motif ranking is found in the region set. The Area Under the Curve (AUC) is normalized based on the average AUC for all motifs and their standard deviation, resulting in a Normalized Enrichment Score (NES) that is used to quantify the enrichment of a motif in a set of regions. For each motif, regions significantly enriched in the foreground based on DEM; and regions found withing the leading edge of the cisTarget ranking are selected as positive hits (see *Methods*). For each TF, we take the union of regions associated with its motifs, resulting in a TF cistrome.

To benchmark the different motif enrichment techniques included in pycisTarget and approaches to build databases, the recovery of target TFs was assessed using 309 ChIP-seq data sets from the Deeply Profiled Cell Lines collection from ENCODE[34,35] that were also included in Unibind[36,37] (Supplementary Table 4). Motif enrichment was performed using Homer, cisTarget and DEM. For the latter two, three different approaches for creating the motif databases were used: 1) generating a database without clustering the motif collection and only retaining annotated motifs (24,309 motifs, named unclustered (u)), 2) generating a database using a single consensus motif (or archetype) for each of the STAMP clusters (named archetype (a)) and 3) generating a database by scoring regions using all the motifs in a cluster (as described above, named clustered (c)). In addition, since our motif collection contains licensed motifs from Transfac Pro, we also benchmarked cisTarget and DEM using a publicly shareable clustered collection (named public (p), using all PWMs for scoring but removing these protected motifs, finding equal TF recovery and comparable scores in regions.

To benchmark the sensitivity of cisTarget and DEM to identify partner TFs between different target regions of the same TF (depending on the cellular context), we compared the SOXE cistromes inferred in melanoma cell lines (SOX10, from the

melanoma case study), oligodendrocytes (SOX10, from the human cortex case study) and astrocytes (SOX9, from the human cortex case study). These cistromes contain 18,506, 2,553 and 6,817 target regions, for melanoma, oligodendrocytes, and astrocytes, respectively. As cisTarget uses as background other regions in the genome (or consensus peaks), motif enrichment analysis of SOXE cistromes in melanoma, oligodendrocytes and astrocytes with these methods mostly identifies highly enriched SOXE motifs (Supplementary Fig. 2i), with NES 31.6, 22.31 and 31.85, respectively. On the other hand, DEM allows to compare sets of regions in a pairwise manner, allowing to directly assess differences between the sets of SOXE target regions. DEM reveals NFI and HOX motifs on SOXE target regions in astrocytes, OLIG (E-box) in oligodendrocytes, and AP1, RUNX, TFAP2 and MITF motifs in melanoma, in agreement with literature (Supplementary Fig. 2j)[31].

Supplementary Note 4: Benchmark of GRN inference methods

While all methods aim to infer gene regulatory networks, there are conceptual differences that result in different types of GRNs and insights (Fig. 3). For instance, SCENIC[38] only uses scRNA-seq, while the remaining methods use (single-cell) multi-omics data as input. CellOracle[39], like SCENIC, only provides TF-Gene networks (despite using chromatin information internally), infers region-gene links only based on accessibility and compared to other methods does not assess repression, but can predict perturbation effects. Pando[40] co-optimizes TF-region-gene relationships, resulting in one unique score for each combination, which hinders the assessment of region-gene relationships as most regions are targeted by more than one TF, resulting in several scores for each region-gene pair. FigR[41] derives DORCs (Domains of Regulatory Chromatin), which consist of sets of regions (50kb from the gene TSS by default) whose accessibility correlates with the gene expression. Motif enrichment is then performed across the whole DORC, which prevents assessing to which region within the DORC a TF is binding. GRaNIE[42] is the only tool conceptually similar to SCENIC+ (consisting of different steps to infer TF-region and region-gene relationships and a final eGRN compilation step), but it is designed for bulk data. As the original ENCODE Deeply Profiled Cell Lines data are bulk profiles, we tested GRaNIE with these bulk data. However, its performance was very poor, only finding 26 TFs and 11,106 TF-region-gene links. Interestingly, when we applied it to our

simulated single-cell data set (used for all the other tools), its recovery increased (finding 39 TFs and 44,666 TF-region-gene links); hence, we report the latter results. Finally, SCENIC+ not only builds enhancer-GRNs (with TF-region and region-gene information), but can also assess repression, regulon specificity, the effect of TF perturbation (as CellOracle) and prioritize eGRNs driving differentiation processes. Importantly, both SCENIC and SCENIC+ use the SCENIC+ motif collection with thousands of motif/clusters, while most of the remaining methods rely on few hundreds of motifs.

Feature	SCENIC	CellOracle	Pando	FigR	GRaNIE	SCENIC+
Input	scRNA-seq	scRNA-seq and bulk/scATAC-seq	scRNA-seq and scATAC-seq	scRNA-seq and scATAC-seq	Bulk RNA-seq and bulk ATAC-seq	scRNA-seq and scATAC-seq
# Motifs	49,054[1]	8,049[2]	1,590	1,141	768	49,054[1]
# TFs (Human)	1,553	1,022	1,372	1,141	681	1,553
TF-Region	No	No[3]	Yes	No	Yes	Yes
Region-Gene	No	Yes	No	Yes	Yes	Yes
TF-Gene	Yes	Yes	Yes	Yes	Yes	Yes
Output GRN	TF-Gene	TF-Gene	TF-Region-Gene	TF-Gene	TF-Region-Gene	TF-Region-Gene
GRN selection	NA	P-value < 0.01	P-value < 0.01	IZ-scoreI > 0.5	FDR < 0.2	Leading edge
Repression	No	No	Yes	Yes	Yes	Yes
Network activity	Yes	No	No	No	No	Yes
Network specifity	Yes	Yes	No	No	No	Yes
Network visuals	No	No	No	Yes	Yes	Yes
Perturbation	No	Yes	No	No	No	Yes
Differentiation	No	No	No	No	No	Yes

We used SCENIC(+) using cisTarget clustered motif collection, with 12,935 clusters[1]
We used CellOracle with gimmemotifs clustered motif collection (default), with 1,795 clusters[2]
This information is not directly provided in the workflow but it is possible to derive it using the internal motif hits in the regions and the selected region-gene and TF-gene relationships[3]

Table comparing state-of-the-art methods for GRN inference at single cell resolution.

SCENIC and FigR were excluded from the TF-region benchmark since they perform motif enrichment in a space around the TSS or DORCs rather than individual regions, respectively. SCENIC and Pando were excluded from the region-gene benchmark since SCENIC does not calculate region-gene relationships and Pando reports a score per TF-region-gene triplet. Because generally several TFs can bind to the same region, this results in several scores for the same region-gene pair.

Of note, we found that knocking down different master regulators in K562 (e.g. STAT5A, HOXB9, HOXB4, SFPQ, GATA1, GATA2, ARID3A) can result in similar effects and affect similar regulons. These downstream effects can be largely explained by indirect effects of the TF-knockdown experiment direct interactions between the TFs (i.e the targeted TF activates that other TF) or cooperativity (i.e. these TFs target the same genes) (Supplementary Fig. 5). Interestingly, we also observed that some

repressive regulons are upregulated upon knockdown for the corresponding TFs, such as HOXB4, HOXB9 and ARID3A[43,44], showing that SCENIC+ is also able to accurately recover repressive interactions (Supplementary Fig. 5).

Supplementary Note 5: Spatial projection of SCENIC+ regulons in the mammalian cortex

To localize the eGRNs identified by SCENIC+ in the mouse cortex, we generated a smFISH atlas using 100 genes (based on marker genes per cell type and literature (see Methods)). We then used Tangram[45] to transfer cell type annotations and gene expression to the segmented cells in the smFISH map (Supplementary Fig. 9e) and scored the SCENIC+ regulons using AUCell on the imputed complete transcriptome. In line with our previous analyses, this shows activity of Rfx3 in the L2/3 area, Cux1 and Mef2c from L2/3 to L4, Rorb in L4, Etv1 in L5, Fezf2 in L5 and L6, Tbr1 in L6, and Sox10 in the white matter (Supplementary Fig. 9f-g).

Additionally, we analyzed a publicly available multiome dataset of the human cerebellum with matched 10x Visium data from 10x Genomics. From the scRNA-seq data of the multiome we annotated nine main cell types, namely OPC, oligodendrocytes, Purkinje cells, granule cells, inhibitory neurons (Vip+, Sst+, Pvalb+ and Sncg+), and Bergman glia (Fig S24d). Despite the small number of cells in this data set (1,736), SCENIC+ identified 111 regulons, including DLX1 and DLX6 in CGE interneurons (80 cells), LHX6 in MGE interneurons (134 cells), NEUROD2 and EGR4 in granule cells (76 cells), NEUROD1 in Purkinje cells (20 cells), PRRX1 and OLIG2 in OPC (157 cells), SOX10 and TCF12 in mature oligodendrocytes (823 cells), SPI1 and RUNX1 in microglia (67 cells), and NFIA and SOX9 in the Bergman glia (276 cells)[46–50]. SCENIC+ identified a novel master regulator of granule cells, namely EMX1. EMX1 has been previously shown to have an effect on the size and morphology of the cerebellum and hippocampus, but to our knowledge, this TF had not been recognized as key regulator of granule cells[51]. These results show that, even in small data sets and for rare populations, SCENIC+ can infer *bona fide* gene regulatory networks (Supplementary Fig. 9h).

We then scored SCENIC+ regulons onto the 10X Visium spots using AUCell. This shows the LHX6 regulon enriched in the molecular layer of the human cerebellum, where interneurons reside; SOX9 on the Purkinje cell layer, where the Bergman glia

are found; EGR4 in the granule cell layer, and SOX10 in the white matter, which is populated by oligodendrocytes (Supplementary Fig. 9j), in agreement with literature[50,52].

Supplementary Note 6: Predicting repressive interactions using SCENIC+

Transcriptional repression is an important biological mechanism mostly studied in the context of developmental biology[53–56], in which transcription factors (TFs) of one cell type repress TFs of another (adjacent) cell type thereby locking in the fate of the first cell type. Repression is mostly analyzed using genetic gain and loss-of-function experiments[53], in which it is difficult to disentangle direct from indirect effects. For this reason, the mechanisms by which transcription factors (TFs) induce repression on a molecular level are poorly understood.

The eukaryotic genome is compacted in chromatin which maintains a restrictive ground state/default “off” state. To activate transcription, (combinations of) TFs bind enhancers[57], displacing nucleosomes and opening up the chromatin thereby lifting the restrictive ground state. In the context of this model in order to induce repression the chromatin has to be closed again. For this, two main molecular mechanisms are described. First, the access of the activator TF(s) to the chromatin can be limited[53]. For this, the cell can simply stop transcribing the activator TF(s); the cell can transcribe a “repressor” TF with a similar DNA binding domain as the activator thereby excluding the activator by going into direct competition for binding the DNA; the activator TF(s) can be sequestered away from the nucleus using protein-protein interactions; or the amount of activator protein can be limited due to repression at the RNA-level. Second, the chromatin can be actively closed using repressor TFs which, upon binding the DNA, recruit repressive co-factors[58].

To detect this type of repression (repression by chromatin closing) SCENIC+ relies on observing negative correlations between on the one hand TF expression and target gene expression and on the other hand region accessibility and target gene expression. A problem with this approach ensues whenever two or more TFs are expressed in the system of interest for which the motif is the same or very similar but the expression is anti-correlated. In this case it is impossible to deduce whether the chromatin is being closed simply by the absence of the activator TF or it is actively closed by the action of the inferred repressor TF (potentially recruiting co-repressors)

(Supplementary Fig. 10a). This problem is most prevalent in species with many paralogs for which there are many TFs belonging to the same family, for example human[59].

We illustrate this issue in the melanoma cell line analysis. In this system, the expression of the following pairs of TFs is anti-correlated but their motifs are very similar: SOX10 and SOX9, MITF and TCF4 and TCF4 and MITF (Supplementary Fig. 10b). Because of this SCENIC+ predicts repressive eRegulons for SOX9, TCF4 and MXI1 (Supplementary Fig. 10c) and the predicted target regions of these TFs strongly overlap with the predicted target regions of their activating partners (Fig. S12d). Whether SOX9, TCF4 and MXI1 actively close, in the mesenchymal state, the regions opened up by respectively SOX10, MITF and TCF4 in melanocytic/intermediate state cannot be concluded by the SCENIC+ analysis on its own. Using SCENIC+ alone both the scenario in which the regions are passively closed simply by the absence of the activators in the mesenchymal state or actively closed by the presence of the repressors in the mesenchymal state has the same likelihood.

Even though one has to be cautious with the interpretation of repressors predicted by SCENIC+, these predictions can still lead to novel insights. An interesting example in the melanoma cell line analysis is HES1. SCENIC+ predicts a repressive eRegulon for HES1, in line with the function of its ortholog in *Drosophila melanogaster*[60]. HES1 is a basic helix-loop-helix transcription factor and thus its DNA binding motif is very similar to MITF however its expression is not exactly anti-correlated to MITF (Supplementary Fig. 10e). MITF and HES1 are co-expressed in MM031, MM011 and all cell lines of the intermediate state (Supplementary Fig. 10e) while MITF but not HES1 is expressed in MM001. Given that the expression of HES1 is a better prediction for the region accessibility of predicted MITF target regions compared to the expression of MITF itself (Supplementary Fig. 10f) and the predicted target regions and genes of HES1 strongly overlap with those of MITF (Supplementary Fig. 10g) we hypothesize that HES1 represses the action of MITF in melanoma cell lines which co-express both factors thus restricting MITF target gene expression to MM001. This is in line with recent reports where Notch signaling is shown to counteract the effect of MITF in melanoma[61] and where HES1 is shown to enhance epithelial-to-mesenchymal transitions[62].

In the eye-antennal disc, the Cut (Ct) transcription factor, expressed in the antennae where it acts as repressor of the eye field, was indeed identified as candidate

repressor, targeting 13 other TFs (e.g., ss, ey, toy and Optix) (Supplementary Fig. 10h-i). While it has been previously shown that Ct is necessary for the development of the antenna by inhibiting the eye fate[63], SCENIC+ shows that Cut works by directly repressing these master TFs for eye development.

Supplementary References

1. Bravo González-Blas, C. *et al.* cisTopic: cis-regulatory topic modeling on single-cell ATAC-seq data. *Nat. Methods* **16**, 397–400 (2019).
2. Blei, D. M. Latent Dirichlet Allocation. 30.
3. Zhang, Y. *et al.* Model-based Analysis of ChIP-Seq (MACS). *Genome Biol.* **9**, R137 (2008).
4. Corces, M. R. *et al.* The chromatin accessibility landscape of primary human cancers. *Science* **362**, eaav1898 (2018).
5. The ENCODE Project Consortium *et al.* Expanded encyclopaedias of DNA elements in the human and mouse genomes. *Nature* **583**, 699–710 (2020).
6. Wolock, S. L., Lopez, R. & Klein, A. M. Scrublet: Computational Identification of Cell Doublets in Single-Cell Transcriptomic Data. *Cell Syst.* **8**, 281-291.e9 (2019).
7. Mimno, D., Wallach, H. M., Talley, E., Leenders, M. & McCallum, A. Optimizing semantic coherence in topic models. in *Proceedings of the Conference on Empirical Methods in Natural Language Processing* 262–272 (Association for Computational Linguistics, 2011).
8. Griffiths, T. L. & Steyvers, M. Finding scientific topics. *Proc. Natl. Acad. Sci.* **101**, 5228–5235 (2004).
9. Arun, R., Suresh, V., Veni Madhavan, C. E. & Narasimha Murthy, M. N. On Finding the Natural Number of Topics with Latent Dirichlet Allocation: Some Observations. in *Advances in Knowledge Discovery and Data Mining* (eds. Zaki, M. J., Yu, J. X., Ravindran, B. & Pudi, V.) vol. 6118 391–402 (Springer Berlin Heidelberg, 2010).
10. Cao, J., Xia, T., Li, J., Zhang, Y. & Tang, S. A density-based method for adaptive LDA model selection. *Neurocomputing* **72**, 1775–1781 (2009).

11. Otsu, N. A Threshold Selection Method from Gray-Level Histograms. *IEEE Trans. Syst. Man Cybern.* **9**, 62–66 (1979).
12. Yen, Fu-Juay Chang, & Shyang Chang. A new criterion for automatic multilevel thresholding. *IEEE Trans. Image Process.* **4**, 370–378 (1995).
13. Li, C. H. & Lee, C. K. Minimum cross entropy thresholding. *Pattern Recognit.* **26**, 617–625 (1993).
14. Van de Sande, B. *et al.* A scalable SCENIC workflow for single-cell gene regulatory network analysis. *Nat. Protoc.* **15**, 2247–2276 (2020).
15. Wolf, F. A., Angerer, P. & Theis, F. J. SCANPY: large-scale single-cell gene expression data analysis. *Genome Biol.* **19**, 15 (2018).
16. Korsunsky, I. *et al.* Fast, sensitive and accurate integration of single-cell data with Harmony. *Nat. Methods* **16**, 1289–1296 (2019).
17. Polański, K. *et al.* BBKNN: fast batch alignment of single cell transcriptomes. *Bioinformatics* btz625 (2019) doi:10.1093/bioinformatics/btz625.
18. Hie, B., Bryson, B. & Berger, B. Efficient integration of heterogeneous single-cell transcriptomes using Scanorama. *Nat. Biotechnol.* **37**, 685–691 (2019).
19. McLean, C. Y. *et al.* GREAT improves functional interpretation of cis-regulatory regions. *Nat. Biotechnol.* **28**, 495–501 (2010).
20. Davie, K. *et al.* A Single-Cell Transcriptome Atlas of the Aging Drosophila Brain. *Cell* **174**, 982-998.e20 (2018).
21. Janky, R. *et al.* iRegulon: From a Gene List to a Gene Regulatory Network Using Large Motif and Track Collections. *PLoS Comput. Biol.* **10**, e1003731 (2014).
22. Imrichová, H., Hulselmans, G., Kalender Atak, Z., Potier, D. & Aerts, S. i-cisTarget 2015 update: generalized cis-regulatory enrichment analysis in human, mouse and fly. *Nucleic Acids Res.* **43**, W57–W64 (2015).

23. Verfaillie, A., Imrichova, H., Janky, R. & Aerts, S. iRegulon and i-cisTarget: Reconstructing Regulatory Networks Using Motif and Track Enrichment. *Curr. Protoc. Bioinforma.* **52**, (2015).
24. Heinz, S. *et al.* Simple Combinations of Lineage-Determining Transcription Factors Prime cis-Regulatory Elements Required for Macrophage and B Cell Identities. *Mol. Cell* **38**, 576–589 (2010).
25. Gupta, S., Stamatoyannopoulos, J. A., Bailey, T. L. & Noble, W. Quantifying similarity between motifs. *Genome Biol.* **8**, R24 (2007).
26. Frith, M. C. Cluster-Buster: finding dense clusters of motifs in DNA sequences. *Nucleic Acids Res.* **31**, 3666–3668 (2003).
27. Aibar, S. *et al.* SCENIC: single-cell regulatory network inference and clustering. *Nat. Methods* **14**, 1083–1086 (2017).
28. Hopfensitz, M. *et al.* Multiscale Binarization of Gene Expression Data for Reconstructing Boolean Networks. *IEEE/ACM Trans. Comput. Biol. Bioinform.* **9**, 487–498 (2012).
29. Suo, S. *et al.* Revealing the Critical Regulators of Cell Identity in the Mouse Cell Atlas. *Cell Rep.* **25**, 1436-1445.e3 (2018).
30. Aerts, S. *et al.* Gene prioritization through genomic data fusion. *Nat. Biotechnol.* **24**, 537–544 (2006).
31. Minnoye, L. *et al.* Cross-species analysis of enhancer logic using deep learning. *Genome Res.* **30**, 1815–1834 (2020).
32. Caiazzo, M. *et al.* Direct conversion of fibroblasts into functional astrocytes by defined transcription factors. *Stem Cell Rep.* **4**, 25–36 (2015).

33. Wißmüller, S., Kosian, T., Wolf, M., Finzsch, M. & Wegner, M. The high-mobility-group domain of Sox proteins interacts with DNA-binding domains of many transcription factors. *Nucleic Acids Res.* **34**, 1735–1744 (2006).
34. The ENCODE Project Consortium. An integrated encyclopedia of DNA elements in the human genome. *Nature* **489**, 57–74 (2012).
35. Luo, Y. *et al.* New developments on the Encyclopedia of DNA Elements (ENCODE) data portal. *Nucleic Acids Res.* **48**, D882–D889 (2020).
36. Puig, R. R., Boddie, P., Khan, A., Castro-Mondragon, J. A. & Mathelier, A. UniBind: maps of high-confidence direct TF-DNA interactions across nine species. *BMC Genomics* **22**, 482 (2021).
37. Gheorghe, M. *et al.* A map of direct TF–DNA interactions in the human genome. *Nucleic Acids Res.* **47**, e21–e21 (2019).
38. Aibar, S. *et al.* SCENIC: single-cell regulatory network inference and clustering. *Nat. Methods* **14**, 1083 (2017).
39. Kamimoto, K., Hoffmann, C. M. & Morris, S. A. *CellOracle: Dissecting cell identity via network inference and in silico gene perturbation*. http://biorxiv.org/lookup/doi/10.1101/2020.02.17.947416 (2020) doi:10.1101/2020.02.17.947416.
40. Fleck, J. S. *et al.* Inferring and perturbing cell fate regulomes in human brain organoids. *Nature* 1–8 (2022) doi:10.1038/s41586-022-05279-8.
41. Kartha, V. K. *et al.* Functional inference of gene regulation using single-cell multi-omics. *Cell Genomics* **2**, 100166 (2022).
42. Kamal, A. *et al. GRaNIE and GRaNPA: Inference and evaluation of enhancer-mediated gene regulatory networks applied to study macrophages*.

http://biorxiv.org/lookup/doi/10.1101/2021.12.18.473290 (2021) doi:10.1101/2021.12.18.473290.

43. Cain, B. & Gebelein, B. Mechanisms Underlying Hox-Mediated Transcriptional Outcomes. *Front. Cell Dev. Biol.* **9**, (2021).
44. Rhee, C. *et al.* Arid3a is essential to execution of the first cell fate decision via direct embryonic and extraembryonic transcriptional regulation. *Genes Dev.* **28**, 2219–2232 (2014).
45. Biancalani, T. *et al.* Deep learning and alignment of spatially resolved single-cell transcriptomes with Tangram. *Nat. Methods* **18**, 1352–1362 (2021).
46. Butt, S. J. B. *et al.* Transcriptional Regulation of Cortical Interneuron Development. *J. Neurosci.* **27**, 11847–11850 (2007).
47. Olson, J. M. *et al.* NeuroD2 is necessary for development and survival of central nervous system neurons. *Dev. Biol.* **234**, 174–187 (2001).
48. Sarropoulos, I. *et al.* Developmental and evolutionary dynamics of cis-regulatory elements in mouse cerebellar cells. *Science* **373**, eabg4696 (2021).
49. BRAIN Initiative Cell Census Network (BICCN) *et al.* A multimodal cell census and atlas of the mammalian primary motor cortex. *Nature* **598**, 86–102 (2021).
50. van Essen, M. J., Nayler, S., Becker, E. B. E. & Jacob, J. Deconstructing cerebellar development cell by cell. *PLoS Genet.* **16**, e1008630 (2020).
51. Kobeissy, F. H. *et al.* Deciphering the Role of Emx1 in Neurogenesis: A Neuroproteomics Approach. *Front. Mol. Neurosci.* **9**, (2016).
52. Leung, A. W. & Li, J. Y. H. The molecular pathway regulating Bergmann glia and folia generation in the cerebellum. *Cerebellum Lond. Engl.* **17**, 42–48 (2018).
53. Delás, M. J. & Briscoe, J. Chapter Eight - Repressive interactions in gene regulatory networks: When you have no other choice. in *Current Topics in*

Developmental Biology (ed. Peter, I. S.) vol. 139 239–266 (Academic Press, 2020).

54. Howard, M. L. & Davidson, E. H. cis-Regulatory control circuits in development. *Dev. Biol.* **271**, 109–118 (2004).
55. Jacob, J. *et al.* Transcriptional repression coordinates the temporal switch from motor to serotonergic neurogenesis. *Nat. Neurosci.* **10**, 1433–1439 (2007).
56. Orkin, S. H. Diversification of haematopoietic stem cells to specific lineages. *Nat. Rev. Genet.* **1**, 57–64 (2000).
57. Long, H. K., Prescott, S. L. & Wysocka, J. Ever-Changing Landscapes: Transcriptional Enhancers in Development and Evolution. *Cell* **167**, 1170–1187 (2016).
58. Payankaulam, S., Li, L. M. & Arnosti, D. N. Transcriptional Repression: Conserved and Evolved Features. *Curr. Biol.* **20**, R764–R771 (2010).
59. Lambert, S. A. *et al.* The Human Transcription Factors. *Cell* **172**, 650–665 (2018).
60. Fisher, A. & Caudy, M. The function of hairy-related bHLH repressor proteins in cell fate decisions. *BioEssays* **20**, 298–306 (1998).
61. Golan, T. & Levy, C. Negative Regulatory Loop between Microphthalmia-Associated Transcription Factor (MITF) and Notch Signaling. *Int. J. Mol. Sci.* **20**, 576 (2019).
62. Rani, A., Greenlaw, R., Smith, R. A. & Galustian, C. HES1 in immunity and cancer. *Cytokine Growth Factor Rev.* **30**, 113–117 (2016).
63. Wang, C.-W. & Sun, Y. H. Segregation of eye and antenna fates maintained by mutual antagonism in Drosophila. *Development* **139**, 3413–3421 (2012).
