# Supplementary material for: SCENIC+: single-cell multiomic inference of enhancers and gene regulatory networks
Source: Nat Methods. 2023 Jul 13;20(9):1355–67. doi: 10.1038/s41592-023-01938-4 (PMC10482700; doi:10.1038/s41592-023-01938-4)
Supplement: Supplementary file 2 — Reporting Summary [file 41592_2023_1938_MOESM2_ESM.pdf]

nature portfolio

Corresponding author(s): Stein Aerts

Last updated by author(s): Apr 21, 2023

Reporting Summary

Nature Portfolio wishes to improve the reproducibility of the work that we publish. This form provides structure for consistency and transparency in reporting. For further information on Nature Portfolio policies, see our Editorial Policies and the Editorial Policy Checklist.

Statistics

For all statistical analyses, confirm that the following items are present in the figure legend, table legend, main text, or Methods section.

n/a	Confirmed	
☐	☒	The exact sample size (*n*) for each experimental group/condition, given as a discrete number and unit of measurement
☐	☒	A statement on whether measurements were taken from distinct samples or whether the same sample was measured repeatedly
☐	☒	The statistical test(s) used AND whether they are one- or two-sided<br>*Only common tests should be described solely by name; describe more complex techniques in the Methods section.*
☐	☒	A description of all covariates tested
☐	☒	A description of any assumptions or corrections, such as tests of normality and adjustment for multiple comparisons
☐	☒	A full description of the statistical parameters including central tendency (e.g. means) or other basic estimates (e.g. regression coefficient) AND variation (e.g. standard deviation) or associated estimates of uncertainty (e.g. confidence intervals)
☐	☒	For null hypothesis testing, the test statistic (e.g. *F*, *t*, *r*) with confidence intervals, effect sizes, degrees of freedom and *P* value noted<br>*Give P values as exact values whenever suitable.*
☐	☒	For Bayesian analysis, information on the choice of priors and Markov chain Monte Carlo settings
☒	☐	For hierarchical and complex designs, identification of the appropriate level for tests and full reporting of outcomes
☐	☒	Estimates of effect sizes (e.g. Cohen's *d*, Pearson's *r*), indicating how they were calculated

Our web collection on statistics for biologists contains articles on many of the points above.

Software and code

Policy information about availability of computer code

Data collection	Motifs from 29 collections were downloaded to build the SCENIC+ motif collection. A description of the collections and references are described in Supplementary Table 3. The SCENIC+ motif collection is available at https://resources.aertslab.org/cistarget/.
Data analysis	PycisTopic (v1.0.1.dev21+g8aa75d8) is available at https://github.com/aertslab/pycisTopic . Pycistarget (v1.0.1.dev17+gd2571bf) is available at https://github.com/aertslab/pycistarget. SCENIC+ (v0.1.dev373+geb5f14e) is available at https://github.com/aertslab/scenicplus. Detailed tutorials and documentation on the SCENIC+ workflow are available at scenicplus.readthedocs.io, while tutorials on pycisTopic and pycistarget (within the SCENIC+ workflow and as standalone packages) are available at pycisTopic.readthedocs.io and pycistarget.readthedocs.io, respectively. Code to generate custom cisTarget databases is available at https://github.com/aertslab/create_cisTarget_databases. Our implementation of Cluster-Buster is available at https://resources.aertslab.org/cistarget/programs/cbust. Notebooks to reproduce the analyses presented in this manuscript are available at https://github.com/aertslab/scenicplus_analyses. Analyses were performed using R 4.0.3 and Python 3.8. Other relevant software and versions: Precrec (v0.12.9), Scrublet (v0.2.3), harmonypy (v0.0.5), Arboreto (v0.1.6), GSEApy (v0.10.8), ctxcore (v0.1.2), umap (v0.5.2), fitsne (v1.2.1), Scikit-Learn (v0.24.2), loomxpy (v0.4.1), Kent (v2.7), networkx (v2.7.1), pyvis (v0.1.3.1), Cytoscape (v3.9.0), Seurat (v4.0.3), STAMP (v1.3), cisTopic (v2.1.0), Scanpy (v1.8.2), pybiomart (v0.2.0), pybigwig (v0.3.18), Signac (v1.3.0), MACS2 (v2.1.2.1), Cicero (v1.6.2), gimmemotifs (v0.17.1), MotifMatchR (v1.10.0), tispec (v0.99.0), UpsetR (v1.4.0), Juicer Tools (v2.13.05), DESeq2 (v1.28.1), cellrager-atac (v2.0.0), cellranger-arc (v2.0.0), bcftools (v1.11), Bowtie2 (v2.4.4), STAR (v2.7.9a) , fastq-mcf (v1.05), deepTools (v3.5.0), matplotlib (v3.5.2), HTseq (v0.9.1), VSN (v0.27.0), CrossMap (v0.6.0), pyGAM (v0.8.0), scVelo (v0.2.5), ScoMAP (v0.1.0), CellProfiler (v4.2.1) Tangram (v1.0.2), ImageJ (v2.3.0/1.53f) and Polylux tool plugin (v1.6.1).

For manuscripts utilizing custom algorithms or software that are central to the research but not yet described in published literature, software must be made available to editors and reviewers. We strongly encourage code deposition in a community repository (e.g. GitHub). See the Nature Portfolio guidelines for submitting code & software for further information.

Data

Policy information about availability of data

All manuscripts must include a data availability statement. This statement should provide the following information, where applicable:

- Accession codes, unique identifiers, or web links for publicly available datasets
- A description of any restrictions on data availability
- For clinical datasets or third party data, please ensure that the statement adheres to our policy

Data generated in this manuscript, namely scATAC-seq in melanoma cell lines, 10x multiome in the mouse cortex and scATAC-seq in the Drosophila eye disc are available in GEO (GSE210749). GRCh38.86 genome annotation used in this study is available on: https://ftp.ensembl.org/pub/release-86/gtf/homo_sapiens/Homo_sapiens.GRCh38.86.chr.gtf.gz, GRCh38 genome index used in this study is available on: https://cf.10xgenomics.com/supp/cell-arc/refdata-cellranger-arc-GRCh38-2020-A-2.0.0.tar.gz, mm10 genome index used in this study is available on: https://cf.10xgenomics.com/supp/cell-arc/refdata-cellranger-arc-mm10-2020-A-2.0.0.tar.gz. Data from ENCODE Deeply Profiled Cell Lines was downloaded from https://www.encodeproject.org/, including bulk RNA-seq and ATAC-seq for 8 cell lines, namely MCF7 (ENCFF136ANW and ENCFF772EFK, for RNA-seq and ATAC-seq, respectively), HepG2 (ENCFF660EXG and ENCFF239RGZ), PC3 (ENCFF874CFD and ENCFF516GDK), GM12878 (ENCFF626GVO and ENCFF415FEC), K562 (ENCFF833WFD and ENCFF512VEZ), Panc1 (ENCFF602HCV and ENCFF836WDC), IMR90 (ENCFF027FUC and ENCFF848XMR) and HCT116 (ENCFF766TYC and ENCFF724QHH); and Hi-C data on 5 of the cell lines (IMR90 (ENCFF685BLG), GM12878 (ENCFF053VBX), HCT116 (ENCFF750AOC), HepG2 (ENCFF020DPP) and K562(ENCFF080DPJ)). STARR-seq data was downloaded from ENCODE (ENCFF045TVA (K562), ENCFF047LDJ (HepG2), ENCFF428KHI (HCT116), ENCFF826BPU (MCF7)). bulk RNA-seq experiments upon perturbation in these cell lines and ChIP-seq data sets are described in Supplementary Table 1 and Supplementary Table 4, respectively. 10x multiome data on PBMC was downloaded from the 10x website. scRNA-seq data of baseline MM-lines and bulk RNA-seq data after SOX10 knockdown were downloaded from GEO (GSE134432). MITF, SOX10 and TFAP2A ChIP-seq data were downloaded from GEO (GSE61965 (MITF and SOX10) and GSE67555 (TFAP2A)). SNARE-seq2 data on the human cortex was downloaded from Bakken et al., 202160. scATAC-seq and scRNA-seq data from the Drosophila eye-antennal disc were downloaded from GEO (GSE115476). 10x Visium data and 10x single cell multiome data from the human cerebellum was downloaded from the 10x website. All analyses can be explored in SCope (https://scope.aertslab.org/#/scenic-v2) and UCSC in the following sessions: PBMC (https://genome-euro.ucsc.edu/s/Seppe%20De%20Winter/scenicplus_pbmc), ENCODE cell lines (https://genome.ucsc.edu/s/cbravo/SCENIC%2B_DPCL), melanoma (http://genome-euro.ucsc.edu/s/Seppe%20De%20Winter/scenicplus_mix_melanoma), mouse and human cortex (https://genome-euro.ucsc.edu/s/cbravo/SCENIC%2B_Cortex), eye-antennal disc (http://genome.ucsc.edu/s/cbravo/SCENIC%2B_EAD) and human cerebellum (https://genome-euro.ucsc.edu/s/cbravo/SCENIC%2B_cerebellum). The SCENIC+ motif collection is available at: https://resources.aertslab.org/cistarget/motif_collections.

Human research participants

Policy information about studies involving human research participants and Sex and Gender in Research.

Reporting on sex and gender	NA
Population characteristics	NA
Recruitment	NA
Ethics oversight	NA

Note that full information on the approval of the study protocol must also be provided in the manuscript.

Field-specific reporting

Please select the one below that is the best fit for your research. If you are not sure, read the appropriate sections before making your selection.

☒ Life sciences ☐ Behavioural & social sciences ☐ Ecological, evolutionary & environmental sciences

For a reference copy of the document with all sections, see nature.com/documents/nr-reporting-summary-flat.pdf

Life sciences study design

All studies must disclose on these points even when the disclosure is negative.

Sample size	No statistical method was used to predetermine sample size. Sample sizes were chosen based on the maximum amount of samples that were available for each analysis (for example, all available ChIP-seq datasets in ENCODE on the Deeply Profiled Cell Lines, used in this study, were used). For each analysis the sample size was sufficient to derive statistically meaningful results passing multiple testing procedures.
Data exclusions	Single cells with low quality on the scRNA-seq (e.g. low number of reads, high percentage of mitochondrial reads) and/or the scATAC-seq data (e.g. low TSS enrichment, FRiP, number of fragments) were excluded, as described in the methods section.
Replication	We provide a Github repository with code to replicate the analyses at: https://github.com/aertslab/scenicplus_analyses
Randomization	Not applicable. Each analysis was independent.

Blinding	Blinding was not relevant to our study as we report an analysis software as main finding.

Reporting for specific materials, systems and methods

We require information from authors about some types of materials, experimental systems and methods used in many studies. Here, indicate whether each material, system or method listed is relevant to your study. If you are not sure if a list item applies to your research, read the appropriate section before selecting a response.

Materials & experimental systems

n/a	Involved in the study
☒	☐ Antibodies
☐	☒ Eukaryotic cell lines
☒	☐ Palaeontology and archaeology
☐	☒ Animals and other organisms
☒	☐ Clinical data
☒	☐ Dual use research of concern

Methods

n/a	Involved in the study
☒	☐ ChIP-seq
☒	☐ Flow cytometry
☒	☐ MRI-based neuroimaging

Eukaryotic cell lines

Policy information about cell lines and Sex and Gender in Research

Cell line source(s)	Patient-derived melanoma cell lines used in this study were obtained from the laboratory of Pr. Ghanem-Elias Ghanem (Istitut Jules Bordet, ULB, Belgium).
Authentication	The identity of each line has been determined using RNA-seq and ATAC-seq.
Mycoplasma contamination	Cell cultures used for experiments providing data to this study were tested for myoplasm contamination and were found to be negative.
Commonly misidentified lines (See ICLAC register)	No commonly misidentified cell lines have been used.

Animals and other research organisms

Policy information about studies involving animals; ARRIVE guidelines recommended for reporting animal research, and Sex and Gender in Research

Laboratory animals	Mice were maintained in a specific pathogen-free facility under standard housing conditions (temperature 20-24C and humidity 45-65%) with continuous access to food and water. Mice used in the study were 57 days old and were maintained on 14 hr light, 10 hr dark light cycle from 7 to 21 hr. In this study, cortical brain tissue from male P57 BL/6Jax was used. Animals were anesthetized with isoflurane, and decapitated. Cortices were collected and immediately snap frozen in liquid nitrogen.
Wild animals	No wild animals were used in this study.
Reporting on sex	Sex is not relevant for this study as we report an analysis software as main finding, therefore sex was not considered in the study design. The findings in this study apply to only one sex (male mice). Sex of mice was determined visually based on anogenital distance and pigmentation.
Field-collected samples	No field collected samples were used in this study.
Ethics oversight	All animal experiments were conducted according to the KU Leuven ethical guidelines and approved by the KU Leuven Ethical Committee for Animal Experimentation (approved protocol numbers ECD P007/2021).

Note that full information on the approval of the study protocol must also be provided in the manuscript.
